# Young microbiome transplantation enhances recovery after myocardial infarction

**DOI:** 10.18632/aging.206279

**Published:** 2025-07-15

**Authors:** Min-Yi You, Tony W.H. Tang, Suminani Novita, Yen-Wen Liu, Kuan-Cheng Chang, Yen-Wen Wu, Yu-Kai Chao, Shu-Chian Ruan, Po-Ju Lin, Hung-Chih Chen, Patrick C.H. Hsieh

**Affiliations:** 1 Institute of Biomedical Sciences, Academia Sinica, Taipei, Taiwan; 2 Division of Cardiology, Department of Internal Medicine, National Cheng Kung University Hospital, College of Medicine, National Cheng Kung University, Tainan, Taiwan; 3 Division of Cardiovascular Medicine, China Medical University Hospital, Taichung, Taiwan; 4 School of Medicine, China Medical University, Taichung, Taiwan; 5 Cardiovascular Medical Center, Far Eastern Memorial Hospital, New Taipei City, Taiwan; 6 Department of Medicine and Stem Cell and Regenerative Medicine Center, University of Wisconsin-Madison, Madison, WI 53706, USA; 7 Institute of Medical Genomics and Proteomics and Institute of Clinical Medicine, National Taiwan University College of Medicine, Taipei, Taiwan

**Keywords:** aging, gut microbiota, myocardial infarction, cardiac repair, metabolites

## Abstract

Background: The relationship between aging, gut microbiota, and cardiac repair after myocardial infarction (MI) remains unclear. Understanding this interaction may provide novel strategies for improving cardiovascular outcomes in the elderly.

Methods: Aged mice were treated with antibiotics followed by fecal microbiota transplantation (FMT) from young or aged donors prior to MI. Cardiac function, gut integrity, immune signaling, and metabolism were evaluated. Gut microbiota and plasma metabolites were also profiled in ST-elevation myocardial infarction (STEMI) patients across age groups.

Results: Young FMT improved post-MI cardiac function and reduced infarct size in aged mice. It preserved intestinal barrier integrity, reduced IL-17A–positive immune cells, and attenuated age-related intestinal shortening. Aging was associated with decreased microbial diversity, loss of beneficial taxa such as *Akkermansia*, and enrichment of inflammatory pathways. Cardiac metabolomics revealed reduced oxidative metabolism and increased lipid reliance in aged mice. In STEMI patients, aging correlated with lower microbiota diversity, altered taxonomic profiles, and shifts in lipid and amino acid metabolism.

Conclusions: This study highlights the role of gut microbiota in cardiovascular health and aging. Microbiota transplantation improved cardiac recovery, suggesting its therapeutic potential and offering new insights into the gut–heart axis for future treatments in age-related cardiovascular disease.

## INTRODUCTION

Aging, a critical risk factor for heart failure (HF) and various cardiovascular diseases (CVDs), brings to the forefront the increasing burden of these conditions in our aging global population [1]. As the incidence of HF escalates from about 0.6-0.79% in individuals aged 45 to approximately 2.1% in those over 65, the correlation between aging and the prevalence of HF becomes glaringly evident [2]. This uptick in HF cases among the elderly, in conjunction with the worldwide trend of an aging population, accentuates the urgency to address these cardiovascular health challenges. Despite modern advancements in HF treatments through guideline-directed medical therapy, there remains a stark and persistent correlation between aging and elevated mortality rates following myocardial infarction (MI), particularly in cases linked with obstructive coronary artery disease [3]. It is, therefore, imperative to focus cardiovascular disease research on the aging population, aiming to discover novel pathogenic mechanisms unique to this demographic.

In recent years, the burgeoning field of microbiome research has revealed a complex network of communication between the gut and various body organs, spotlighting the gut microbiome as a key player in health and disease [4–6]. Age-related changes in the composition, diversity, and functionality of the microbiota, alongside their association with the onset of CVD, have been increasingly recognized [7–9]. The gut microbiota is implicated in the onset of atherosclerosis [5, 10] and modulating cardiac repair in HF [11, 12], primarily through influence of its metabolites on host immune system homeostasis [13]. Imbalance of gut microbiota exacerbates gut permeability and triggers systemic inflammation, particularly in the context of aging [14] and disease [15, 16]. Interventions that address dysbiosis, by restoring gut integrity and immune balance, have shown promising results in disease recovery and lifespan extension [14, 16–18]. With this understanding, interventions targeting the gut-organ axis, such as fecal microbiome transplantation (FMT) and antibiotic therapy, emerge as potential therapeutics in influencing CVD outcomes [16, 19]. However, the effectiveness of such interventions in enhancing post-infarction cardiac repair in the elderly is not yet fully understood.

In this study, we aimed to assess the impact of young microbiome transplantation on cardiac repair after MI in aged mice, compared to older microbiome from older counterparts. We employed 16S V3-V4 NGS and LC-MS to analyze microbial composition and metabolic profile. We further extended our investigation to human STEMI patients, examining gut microbiota and plasma metabolites across different ages. These integrated studies offer valuable insights into the gut-heart axis, particularly in the aging context, and hold potential for developing novel therapeutic strategies for cardiovascular diseases in elderly populations.

## RESULTS

### Replacement of young microbiota in aged mice improved post-infarction cardiac repair

**Figure 1:**
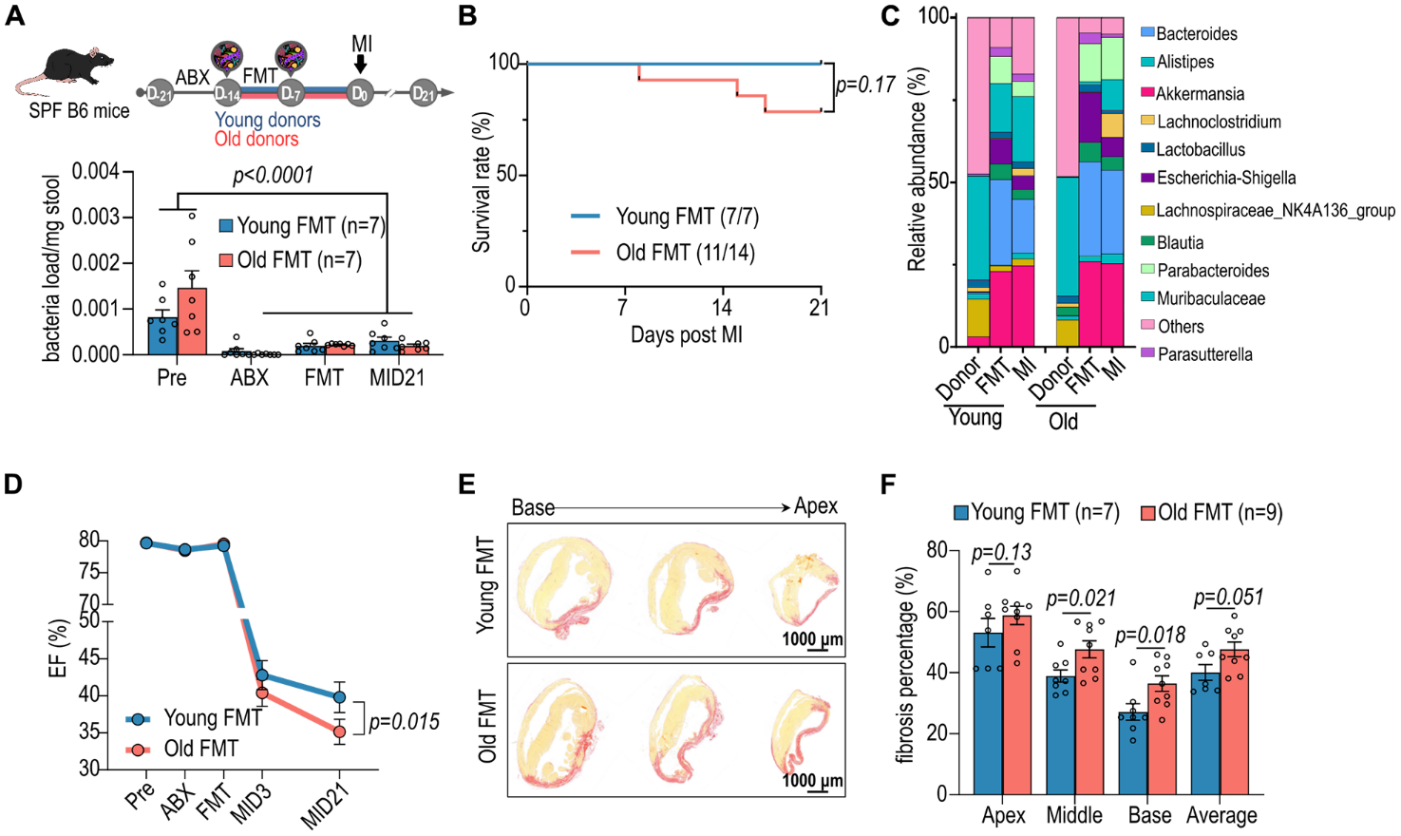
**Impact of aged fecal microbiome transplantation (FMT) on cardiac function in recipient mice.** (**A**) Female specific pathogen-free (SPF) mice were administrated with antibiotics (ABX) and subsequently subjected to FMT from young and aged mice prior myocardial infarction (MI) (upper panel). The lower panel illustrates the bacterial load in the stools of female mice following FMT. (**B**) The post-MI survival curve for female mice subjected to FMT from young and aged mice. (**C**) Post-FMT abundance of top 11 bacteria genera in female mice. (**D**) Cardiac ejection fraction (EF, %) of female mice before and after FMT and MI. (**E**) Picrosirius red staining for post-MI cardiac fibrosis of female mice receiving FMT. (**F**) Statistical analysis of post-MI cardiac fibrosis of female mice receiving FMT. Two-way ANOVA with Tukey post-hoc test was used to analyze data in A, D and F. Data are represented as mean ± SEM.

To investigate the influence of microbiome on post-infarction cardiac repair in aged SPF mice (approximately eighteen months old), the aged female mice were treated with an antibiotic cocktail to deplete the original gut microbiome. Subsequently, sex-matched healthy young microbiome (approximately three months old) was supplemented to the antibiotic-treated mice fourteen days prior to myocardial infarction (MI) induced by left anterior descending (LAD) coronary artery ligation for twenty-one days (Figure 1A). On day 21 post-infarction, there was no significant difference in overall survival between the groups (Figure 1B). However, 16S V3-V4 NGS revealed alterations in the gut microbiome (Figure 1C). Notably, aged mice receiving young microbiome exhibited less decline in ejection fraction (EF) twenty-one days post-MI compared to mice transplanted with aged fecal microbiome (Figure 1D). Additionally, the infarct sizes in both the middle and base sections of the ischemic region were reduced in mice transplanted with young individual-derived microbiome following MI (Figure 1E, 1F). These findings highlight the dependency of improved post-infarction cardiac repair in aged SPF mice, regarding heart function preservation and reduction in cardiac infarction size, on the young gut microbiota. Consistent with the findings in females, male recipients of young microbiota demonstrated improved cardiac function post-MI, with preserved ejection fraction and reduced fibrotic remodeling compared to those receiving aged donor microbiota (Supplementary Figure 1). These findings indicate that the cardioprotective impact of young microbiota is not restricted to a single sex, aligning with prior reports that gut microbial modulation can influence cardiac outcomes in both male and female mice.

### Post-infarction intestinal health was preserved by young microbiome

**Figure 2:**
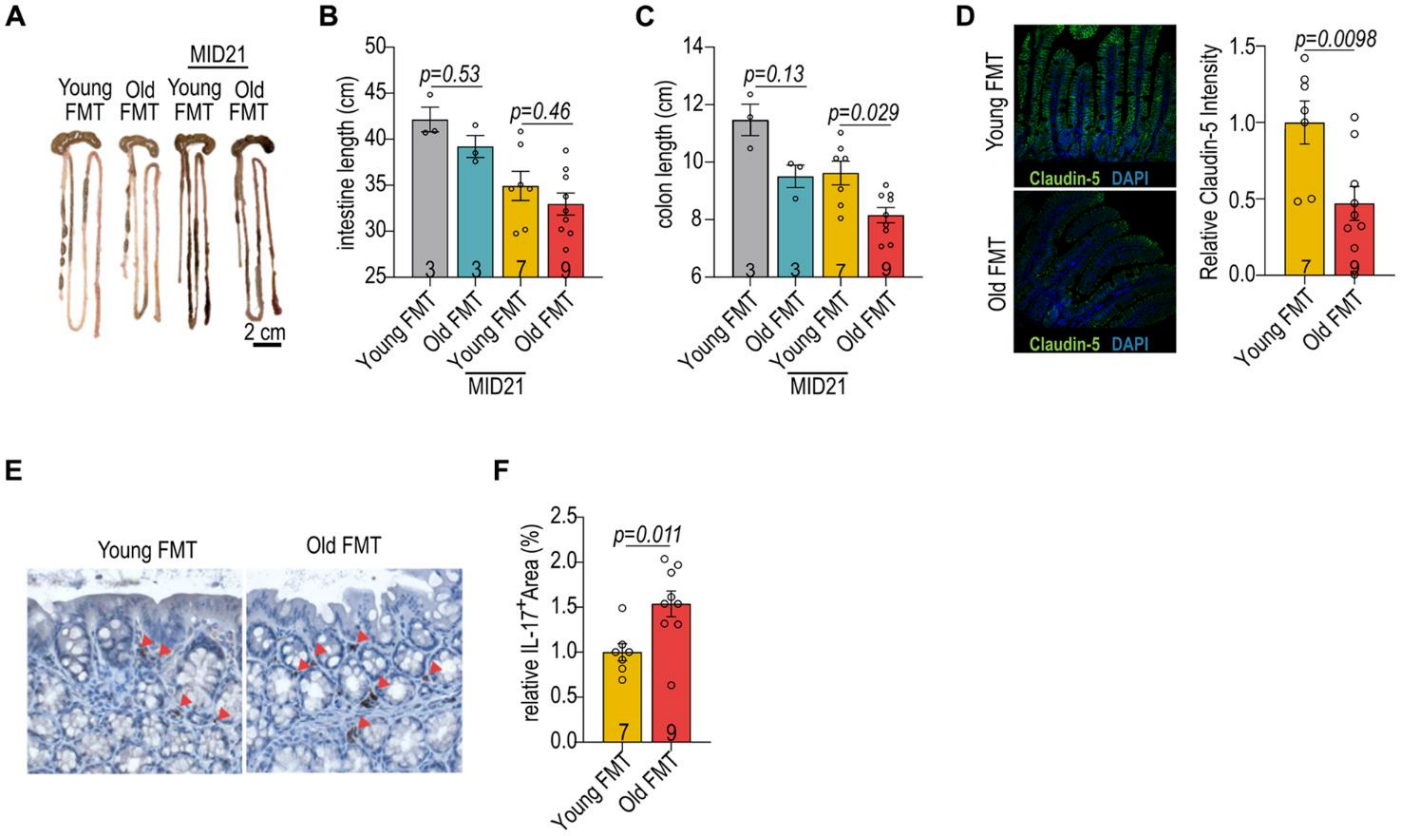
**Impact of age-associated microbiome on gut homeostasis.** (**A**) The gut morphology of aged female mice with FMT from young and old donors. (**B**) The intestinal length of female mice with reciprocal FMT. (**C**) The colon length of female mice with reciprocal FMT. (**D**) Immunofluorescent staining for claudin-5 in the intestines of female mice with reciprocal FMT (left panel). Relative *claudin-5* expression in intestines of female mice with reciprocal FMT (right panel). (**E**) Immunohistochemical staining of IL-17A in the intestines of female mice with reciprocal FMT. (**F**) Quantification of IL-17A^+^ cells in the intestines of female mice with reciprocal FMT. Data in (**B**, **C**) were analyzed with Kruskal–Wallis followed by Dunn’s correction; data in (**D**, **F**) were analyzed with the two-sided Student’s *t*-test. Data are represented as mean ± SEM.

Given that the intestine acts as a barrier between host and bacteria, and circulation and plays a role in systemic inflammation in both diseased and aging states [20], we examined the effects of age-associated microbiome on intestinal morphology. The lengths of small intestine and colon in both recipient groups decreased at post-MI day 21 (Figure 2A–2C). Notably, recipients of old microbiome exhibited a more pronounced decrease in colon length compared to recipients of young microbiome (Figure 2A–2C). Expression of the intestinal tight junction protein Claudin5, which regulates intestinal barrier integrity, was preserved in the intestinal villi of the young microbiome recipient group (Figure 2D). Previous studies have reported higher inflammation and loss of intestinal barrier function in mice transplanted with microbiome from aged donors [21, 22]. Gut microbes play a pivotal role in shaping the local immune system by interacting with various immune cells in the lamina propria [20, 23]. The cells positive for the pro-inflammatory cytokine IL-17A reduced in the colon lamina propria of aged MI mice receiving young microbiome transplantation (Figure 2E, 2F). This finding further supports that gut microbiome derived from the young donors maintain gut healthiness in aged mice post-MI.

### Reduced gut microbial diversity during aging

**Figure 3:**
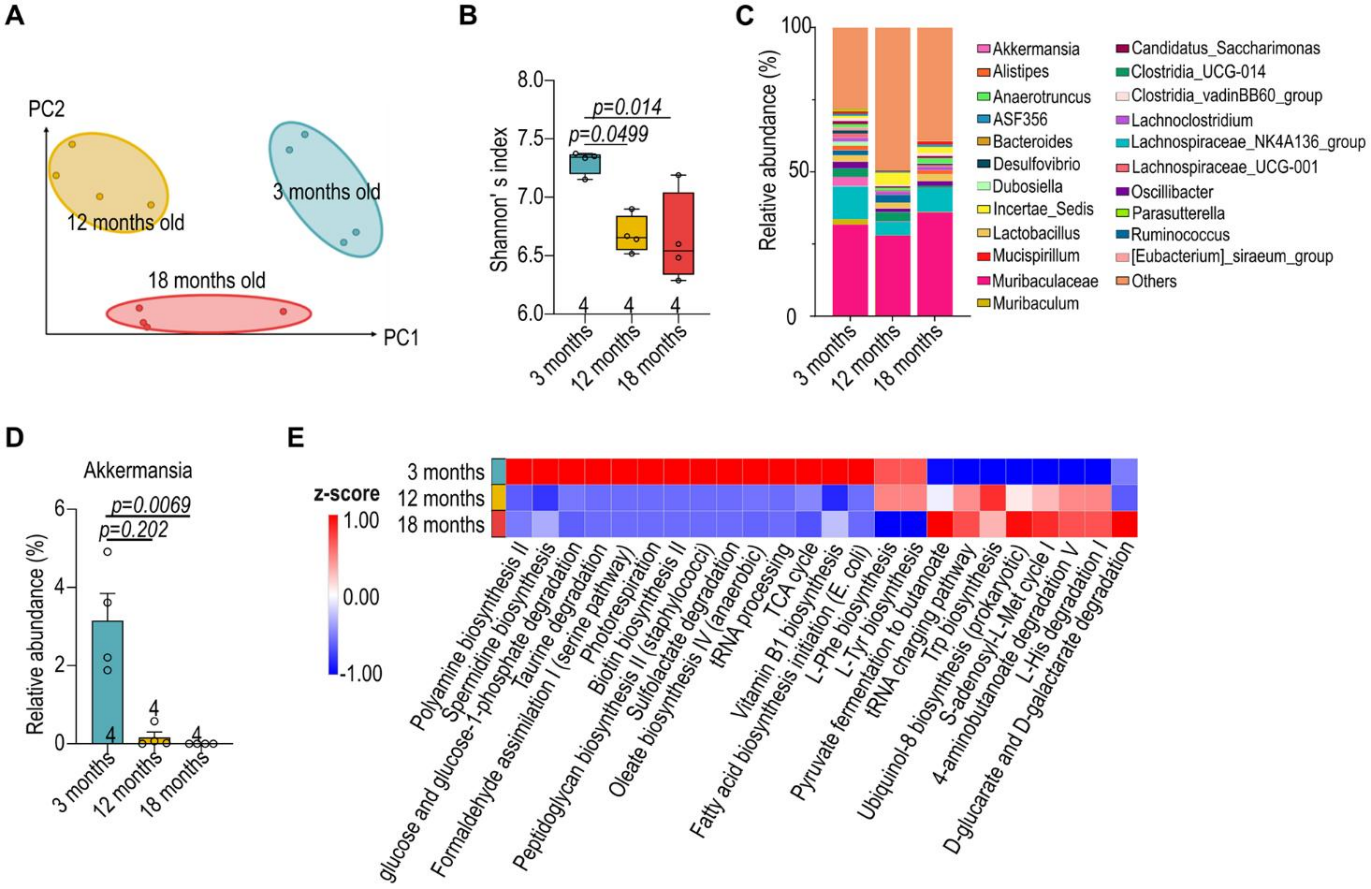
**Reduced diversity in aged microbiota of female B57Bl/6 mice.** (**A**) Weighted UniFrac distance analysis depicting the variation in gut microbial composition at 3-, 12- and 18-month-old female C57BL/6 mice. (**B**) Shannon’s index of female C57BL/6 gut microbiota across different age groups. (**C**) Relative abundance of the top 22 genera in the gut microbiota of male mice at various ages. (**D**) The relative abundance of *Akkermansia*. (**E**) Prediction of differential metabolic pathways with Phylogenetic Investigation of Communities by Reconstruction of Unobserved States (PICRUSt). Data in (**B**, **D**) were analyzed with Kruskal–Wallis followed by Dunn’s correction. Data are represented as mean ± SEM.

To investigate the impact of aging on the gut microbiota, we analyzed variations of the microbial composition and predicted metabolic functions. Weighted UniFrac distance analysis revealed distinct clusters corresponding to each age group, with noticeable dispersion indicating a broadening diversity at three months that gradually narrowed by eighteen months (Figure 3A). The Shannon diversity index, employed to quantify microbial alpha diversity, also demonstrated a decrease in diversity as mice aged from three to eighteen months (Figure 3B). The diverse in gut microbiota alongside age also reflected from the dynamic fluctuations in the top 22 genera in the gut microbiota at various ages, such as an age-dependent constant decrease of *Akkermansia* (Figure 3C, 3D). To gain further insights into the functional implications of age-related microbiota changes, we employed Phylogenetic Investigation of Communities by Reconstruction of Unobserved States (PICRUSt) to predict differential metabolic pathways (Figure 3E). The aged mice showed an increase in the relative abundance of microbial genes involved in the metabolism of compounds that may be harmful; conversely, there was a decrease in genes associated with the metabolism of plant-derived dietary compounds. These changes in microbial metabolism may influence nutrient availability, immune system modulation, and overall energy homeostasis of the host.

### Alterations in cardiac metabolism with age

**Figure 4:**
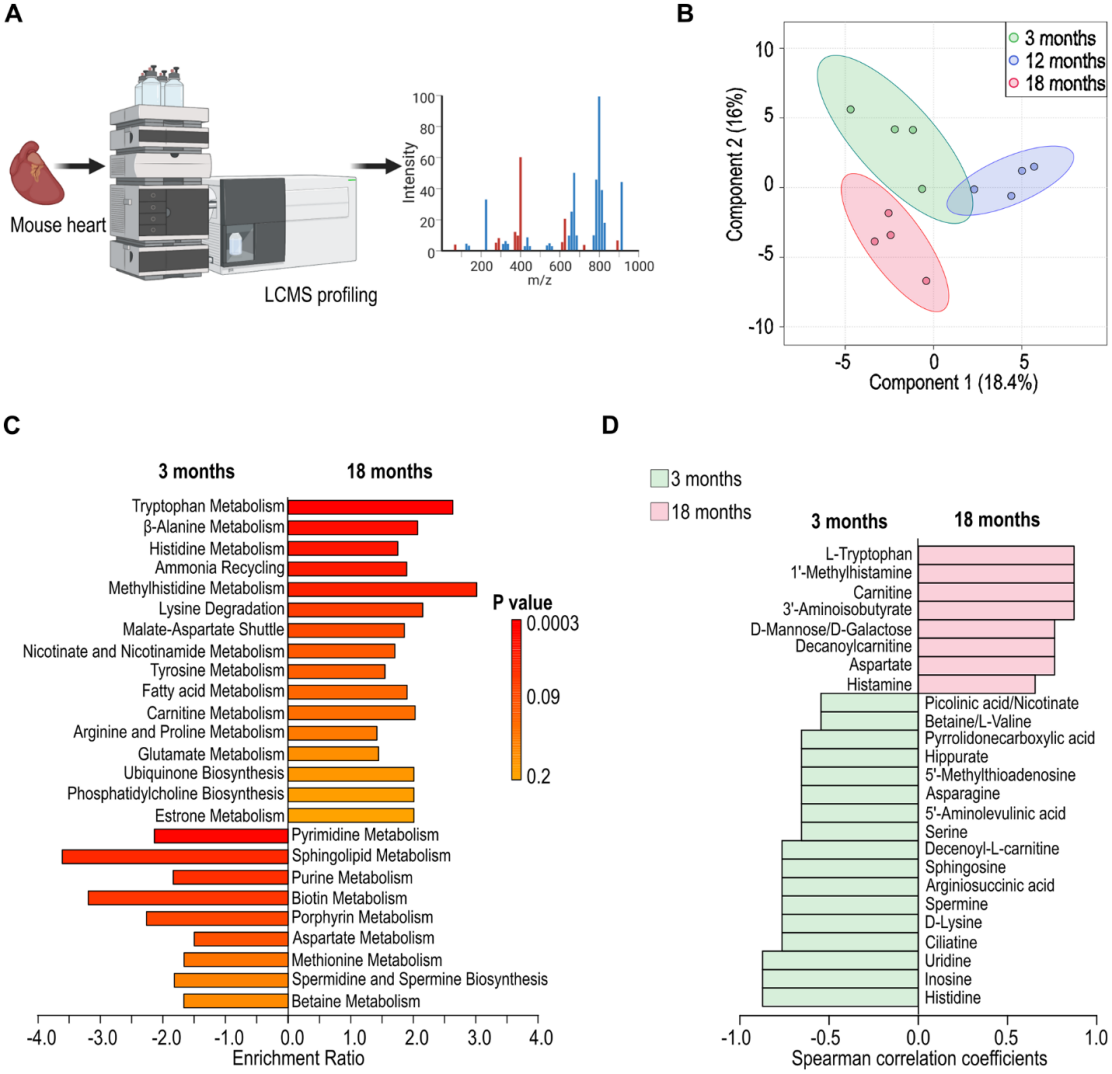
**Alteration in cardiac metabolism in aged female C57BL/6 mice.** (**A**) Schematic illustration of cardiac metabolism assessed with liquid chromatography mass spectrometry (LC-MS). (**B**) Partial Least Squares Discriminant Analysis (PLS-DA) depicting the variation in cardiac metabolites across different ages (3-, 12-, and 18 months). (**C**) Identification of differential metabolic pathways in cardiac tissues at 3- and 18-month of age. (**D**) Analysis of differential cardiac metabolites presented at 3- and 18-month of age.

To identify variations in cardiac metabolites across three age groups, we conducted liquid chromatography mass spectrometry (LC-MS) to assess cardiac metabolism at various ages (Figure 4A). Partial Least Squares Discriminant Analysis (PLS-DA) revealed distinct clusters corresponding to each age group, indicating age-specific cardiac metabolic profiles (Figure 4B). A comparative analysis between 3-month-old and 18-month-old mice exhibited age-associated variations in cardiac metabolism, including those involved in fatty acid metabolism, amino acid degradation, and oxidative stress response, (Figure 4C). Further analysis revealed a notable decrease in metabolites associated with energy production pathways, such as glycolysis and the tricarboxylic acid cycle, alongside an increase in metabolites related to lipid metabolism in mice of eighteen months old (Figure 4D). This shift could reflect a compensatory mechanism or a response to the altered energetic demands of the aging cardiac tissue.

### Alterations in gut microbial composition in ST-elevation myocardial infarction (STEMI) patients across different ages

**Figure 5:**
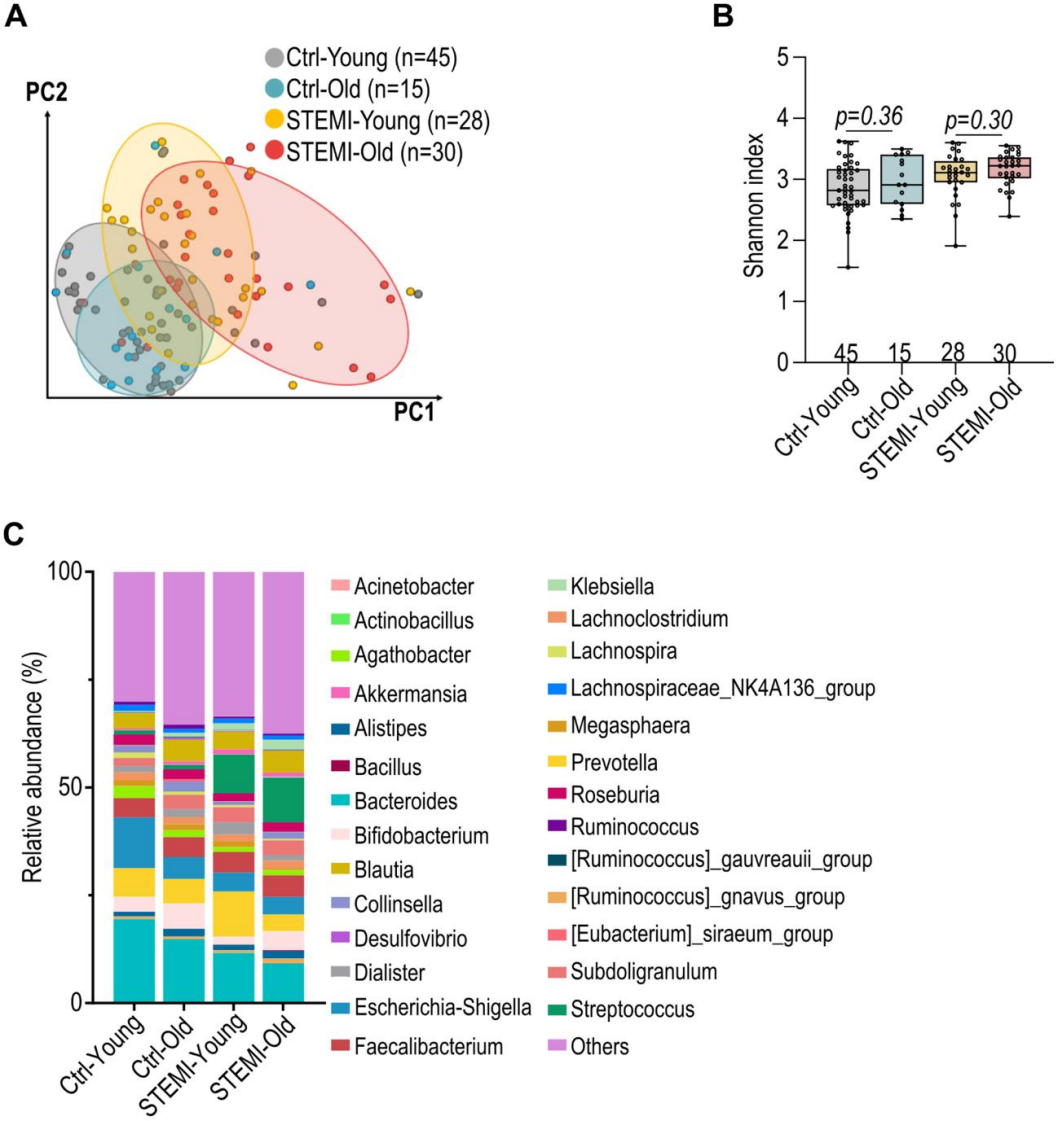
**Alteration in control and ST-elevation myocardial infarction (STEMI) human gut microbiota across various ages.** (**A**) Weighted UniFrac distance analysis illustrating the dissimilarity in control and STEMI human gut microbiota across various age groups. (**B**) Shannon’s index of healthy and STEMI human gut microbiota at varying ages. (**C**) Relative abundance of the top 27 general in normal and STEMI human gut microbiota at different age intervals. Data in (**B**) was analyzed with Kruskal–Wallis followed by Dunn’s correction. Data are represented as mean ± SEM.

Next, we evaluated the dissimilarities in the gut microbiota of individuals with and without ST-elevation myocardial infarction (STEMI) across various age groups. Both control and STEMI groups show distinct microbiota profiles with age (Figure 5A). Notably, the STEMI-affected groups, both young and old, demonstrated a greater variance in microbiota composition compared to their respective control groups (Figure 5A). Alpha diversity of the gut microbiota (Shannon’s index) was affected by the presence of STEMI, with STEMI patients typically showing a reduced alpha diversity compared to control group (Figure 5B). Moreover, the age-related changes in alpha diversity were more pronounced in STEMI patients than in the control groups (Figure 5B). Additionally, a clear shift in the microbial composition was presented between the young and aged groups within both control and STEMI patients (Figure 5C).

### Age-related alterations in human plasma metabolic profiling in STEMI patients

**Figure 6:**
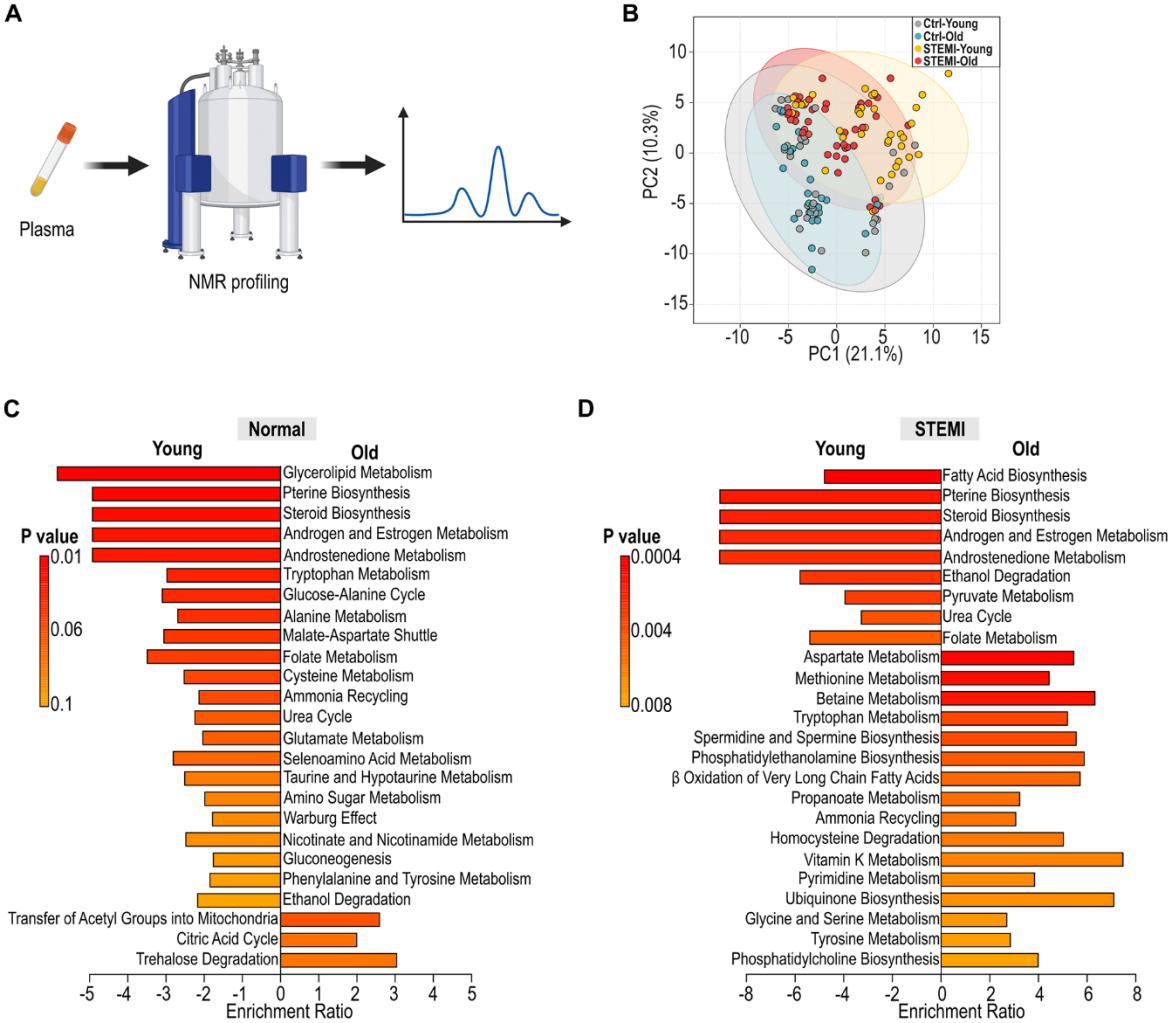
**Alteration in human plasma metabolic profiling across age groups.** (**A**) Schematic illustration of human plasma profiling using NMR. (**B**) PCoA displaying the variation in normal and STEMI human plasma metabolites across different age groups. (**C**) Identification of differential metabolic pathways in young and aged control plasma. (**D**) Analysis of differential metabolic pathways in young and aged STEMI plasma.

To assess metabolic differences between healthy and STEMI patients across different ages, human plasma samples were subjected to nuclear magnetic resonance spectroscopy (NMR) (Figure 6A). Principal coordinates analysis (PCoA) indicating a divergence in metabolic profiles with age, particularly between control and STEMI patients. (Figure 6B). In plasma of control individuals, the metabolic pathways that changed with age included those related to lipid metabolism, amino acid turnover, and antioxidant response, among others (Figure 6C). Notably, the aging process in the control group was characterized by a distinct downregulation of certain metabolic pathways, potentially indicating a reduced metabolic plasticity or an adaptive response to aging. Conversely, in STEMI plasma, there were downregulation in fatty acid biosynthesis as well as upregulation in amino acid metabolisms (Figure 6D). These upregulated pathways in the aged STEMI group suggested an altered metabolic response to the myocardial infarction, which may be related to the pathophysiology or compensatory mechanisms of the aging heart. The distinct patterns observed suggest that aging influences host metabolism differently in the context of STEMI, highlighting potential pathways that could be implicated in the pathophysiology or progression of the condition.

## DISCUSSION

The interconnection between aging, cardiovascular health, and the gut microbiota is increasingly recognized as a determinant in the pathogenesis of age-related diseases. In this study, we focused on evaluating the effects of young microbiome transplantation on post-MI cardiac repair in aged mice, in comparison to age mice receiving older microbiome transplantation. We further extended to human STEMI patients, where we examined gut microbiota and plasma metabolites across various ages. These comprehensive studies shed light on the gut-heart axis, especially in the context of aging, and could pave the way for new therapeutic approaches for cardiovascular diseases in the elderly.

In this study, we initially focused on aged female mice based on previous findings that female mice exhibit more pronounced age-associated changes in gut barrier integrity, immune activation, and microbial composition compared to males [14, 24]. These features made them a suitable model for detecting microbiota-mediated interventions. Nonetheless, to address concerns regarding sex-specific effects, we replicated the FMT protocol in aged male mice and observed comparable improvements in post-MI cardiac function and fibrosis reduction (Supplementary Figure 1). These results suggest that the therapeutic benefit of young microbiota transplantation is likely conserved across sexes. However, sex-dependent differences in microbiota–immune–metabolic interactions remain underexplored [13], and future studies with larger male cohorts and mechanistic analysis will be essential to fully delineate these interactions.

The cardio-protective effects of microbiome derived from young donors (Figure 1D–1F) suggest that gut microbiome could be a key factor in cardiac remodeling post-MI. This aligns with a parallel study, showing improved post-stroke recovery in aged mice with youth fecal transplants [16]. Additionally, shortening of colon length in the mice receiving older FMT is one of the indicators in the assessment of intestinal inflammation [25]. Aged microbiome stimulated an increase in IL-17 producing cells in the lamina propria, particularly notable on day 21 post-MI (Figure 2E, 2F). The production of IL-17 by T cells is known to be modulable by short-chain fatty acids (SCFAs) in the gut, suggesting that manipulating SCFA levels could influence immune homeostasis [24]. Therefore, the composition of microbiota and its metabolites, such as SCFAs, might be pivotal in shaping the immune landscape and consequently affecting the recovery process post-cardiac injury.

The marked reduction in microbial diversity observed in aged mice (Figure 3B) echoes similar patterns in the human elderly population, emphasizing the potential universality of these changes across species [26]. Moreover, our observations of altered microbial communities and their possible implications for host metabolism and disease aligns with the role of gut microbiome in modulating healthy aging [27]. The observed alterations in the abundance of the top 22 genera across different ages could have profound systemic implications (Figure 3C), particularly in metabolic processes and immune function. These shifts may influence the synthesis of vital compounds, such as short-chain fatty acids, which have been shown to play roles in inflammation and gut barrier function [21, 24]. The loss of certain microbial species, such as *Akkermansia muciniphila*, was associated with negative health outcomes such as impaired intestinal integrity and insulin resistance (Figure 3D) [28]. Age-related changes in the gut microbiota composition, such as a decrease in *Clostridiales* and *Bifidobacterium* with an enrichment in *Proteobacteria* and pathobionts like *Enterobacteriaceae*, are characteristic of the dysbiosis observed with aging [29, 30]. This dysbiosis is thought to contribute to inflammation and the decline in immune surveillance, potentially driving host aging.

The PICRUSt prediction provided insights into how age-related changes in the microbiota could contribute to various aspects of host aging, including energy homeostasis, oxidative stress, and immune regulation (Figure 3E). While the decline in microbial diversity with age is well-documented [26, 27], our study extends this observation by linking specific taxonomic shifts to functional alterations in metabolic pathways (e.g., increased lipid metabolism genes), immune responses (elevated colonic IL-17A+ cells), and organ-level outcomes (post-MI ejection fraction and cardiac fibrosis), highlighting the systemic implications of age-associated dysbiosis. Importantly, we demonstrate that these microbial and functional changes are not merely correlative but modifiable, as transplantation of a young microbiota reversed many of these age-associated features in aged hosts. This finding reinforces the causative contribution of the aged microbiome to impaired recovery after MI. Moreover, the parallel age-related microbial and metabolic changes observed in STEMI patients (e.g., loss of *Akkermansia* and SCFA-associated taxa, altered lipid/amino acid pathways) underscore the translational relevance of our murine findings. Thus, our study provides a multi-layered framework linking gut dysbiosis to impaired host resilience in aging, integrating compositional, immunological, metabolic, and physiological data across species.

Moreover, the altered cardiac metabolite profiles and the differential metabolic pathways identified in aged mice suggest an intricate link between metabolic processes and aging, underpinning the development of age-associated cardiac dysfunctions (Figure 4C, 4D). The identified metabolic pathways suggest a decline in the efficiency of energy production, particularly in the form of decreased fatty acid oxidation and increased reliance on anaerobic glycolysis, suggesting a link between gut microbiota and host metabolisms. The shift towards glycolysis is often associated with a hypoxic environment and may lead to an accumulation of lactate and subsequent acidosis, potentially exacerbating myocardial stress [31]. The metabolic inflexibility observed in the 18-month-old mice suggests a diminished ability to switch between fuel sources (Figure 4C), a condition previously implicated in the development of age-related cardiac diseases, including heart failure [1]. Furthermore, alterations in lipid metabolites can be linked to the progression of lipid-related disorders, such as atherosclerosis [32].

Our current study elucidates a notable alteration in the *Bacteroidaceae* family across different age groups, and a significant reduction in the *Lachnospiraceae* family within older STEMI patients (Figure 5C). These changes parallel observations in individuals with frailty, inflammatory gut diseases, and atherosclerosis, underscoring potential microbial markers of health status [7, 33–36]. The *Lachnospiraceae* family, a central constituent of the gut microbiome, is known for its role as a major producer of SCFAs, which play a critical role in modulating immune cell function and facilitating post-injury repair [37]. The observed down-regulation of this SCFA-producing family in older STEMI patients aligns with a shift in the gut’s immune environment [11, 16]. Moreover, we also provide evidence that the observed chronological shifts in genera like *Bacteroides*, *Bifidobacterium*, *Blautia*, and *Akkermansia* are conserved across species, signifying a natural progression in the microbiota composition with age (Figure 5C).

The human aging metabolic signatures characterized by changes in lipid metabolism, amino acid turnover, and oxidative stress response pathways highlight potential biomarkers and therapeutic targets for age-related metabolic dysregulation (Figure 6C). The analysis of differential metabolic pathways in young versus aged STEMI plasma further elucidates the impact of aging on the metabolic response to myocardial ischemia (Figure 6D). The distinct patterns in the metabolic pathways between these two demographics indicate that age profoundly influences the plasma metabolome in STEMI. Furthermore, the differences in the metabolic response to STEMI between age groups may reflect variations in disease severity, therapeutic outcomes, and recovery processes. We recognize that the current cohort size limited stratified analyses by sex or comorbidities. While our study design focused on age-related microbial and metabolic changes in STEMI patients, it was not sufficiently powered to support subgroup analysis based on sex, medication use, or metabolic disease burden. Nevertheless, we observed consistent trends in age-associated reductions in microbial diversity and shifts in metabolic profiles, which parallel those seen in our murine model. These findings suggest that aging is a dominant driver of gut–host remodeling in the post-MI setting. Future large-scale studies will be required to assess the interaction between age, sex, and cardiovascular risk in shaping gut–metabolic profiles. Such efforts may also help identify sex-specific microbial signatures or metabolic responses that influence cardiovascular outcomes and therapeutic response, particularly in older populations.

Although the murine and human datasets were generated separately, they are conceptually linked through the shared observation that aging alters gut microbiota composition, barrier integrity, and metabolic outputs. In aged mice, transplantation of young microbiota improved cardiac recovery, preserved intestinal structure, and reshaped local immune responses (Figures 1, 2). These changes were accompanied by shifts in microbial diversity and predicted metabolic function (Figure 3), as well as altered cardiac metabolism (Figure 4). Similar age-dependent changes were also observed in STEMI patients, including reduced microbial diversity and alterations in SCFA-producing taxa such as *Akkermansia* and *Lachnospiraceae* (Figure 5), and differential plasma metabolic profiles in lipid and amino acid pathways (Figure 6). The consistency between species supports the existence of a conserved aging-associated gut–heart axis.

To address causality, we included an isochronic FMT control in which aged mice received microbiota from aged donors. This comparison (Figure 1D–1F) allowed us to distinguish the specific effects of young versus aged microbiota on cardiac function and fibrosis under the same antibiotic pre-conditioning. Mice receiving young microbiota showed significantly higher post-MI ejection fraction and reduced fibrotic area, suggesting that the observed benefits were attributable to microbial composition rather than antibiotic treatment alone. While an antibiotics-only control group was not included in this study, both FMT groups received identical ABX pre-treatment, minimizing confounding. Moreover, previous studies have demonstrated that ABX alone impairs post-MI immune response and cardiac repair [11], and promotes systemic inflammation in aged hosts [14]. Our current findings, reinforced by microbial and metabolic changes in both mice and STEMI patients (Figures 3–6), support a model in which young microbiota transplantation actively modulates gut–immune–cardiac interactions. While our design controlled for antibiotics by using the same ABX regimen across FMT groups, we recognize that ABX alone may induce transient systemic changes. Future studies including an ABX-only or germ-free group will help to further distinguish microbiota depletion from compositional replacement.

Microbiota transplantation in this study was performed prior to myocardial infarction (MI), constituting a preventive intervention model. This design allowed us to explore whether a young microbial environment could improve host resilience and modulate early inflammatory responses following ischemic injury. However, we recognize that this does not fully reflect clinical scenarios, where patients typically seek treatment after MI onset. While the current findings emphasize the protective potential of pre-existing microbial composition (Figure 1D–1F), they also lay a foundation for investigating whether post-MI modulation of the microbiota could offer therapeutic benefits. Indeed, previous studies have demonstrated that gut-targeted interventions, including short-chain fatty acid supplementation and microbial metabolites, can enhance recovery even when administered after ischemic injury [11, 16]. Future studies will evaluate whether delayed microbiota transplantation or targeted metabolic modulation post-MI can reproduce similar benefits, thereby expanding the translational potential of these findings.

In conclusion, our extensive research approach, starting with observations in microbiome transplantation in rodent models to human STEMI patients, showed the age-dependent effect of microbiome on cardiac protection after injury. This comprehensive investigation not only confirms previous studies but also unravels new aspects of the gut-heart axis, especially in the context of aging. These findings pave the way for innovative therapeutic approaches targeting gut microbiota to improve cardiovascular health in the elderly, offering a novel perspective on managing age-related cardiovascular diseases.

## MATERIALS AND METHODS

### Human sample collection

The stool and plasma samples were collected from patients admitted to National Cheng-Kung University Hospital (NCKUH) from January 2018 to April 2021 (south Taiwan), China Medical University Hospital (CMUH) from March 2019 to April 2021 (central Taiwan), and Far Eastern Memorial Hospital (FEMH) from May 2019 to April 2021 (north Taiwan), Taiwan. The diagnosis of ST-elevation myocardial infarction (STEMI) status was confirmed through electrocardiography and catheterization by board-certified cardiologists at these three medical centers in Taiwan. Excluded from the study were pregnant female patients and those with a history of cancer, organ transplantation, previous myocardial infarction, or decompensated heart failure within the past five years. Stool and plasma samples were collected after primary percutaneous coronary intervention (PCI) and between fourteen to thirty days after the intervention. Collection of the samples and clinical information was approved by the institutional review boards of the participating institutes (IRB on Bio-medical Science Research Academia Sinica, AS-IRB02-110151; Institutional Review Board NCKUH, 8800-4-03-005; Research Ethics Review Committee CMUH, CMUH108-REC3-016(CR-2); Research Ethics Review Committee FEMH, 107175-E). All participants provided informed consent.

### Animals

C57BL/6 specific pathogen-free (SPF) mice were sourced from the National Laboratory Animal Center (NLAC), Taiwan. These mice were maintained in a controlled environment with a 12-hour light/dark cycle and had unrestricted access to sterile food (chow diet; Cat No. 5053; LabDiet, USA) and water. All mouse experiments conducted in this study received approval from both the Academia Sinica Institutional Animal Care and Use Committee and the NLAC Animal Care and Use Committee (IACUC No. 18041211). The surgeon performing the experiments was blinded to the experimental groups, and mice from different experimental groups were assigned to the surgeon in a randomized manner.

### Surgery and echocardiography

Myocardial infarction (MI) was induced by permanently ligating the left anterior descending coronary artery, a procedure performed 2 to 3 mm distal to the left atrial appendage, as previously detailed [11]. After 21 days following MI induction, cardiac function was evaluated through echocardiography using a Vivid-q Ultrasound system equipped with a 5- to 13-MHz intraoperative probe from GE.

### Fecal microbiome transplantation (FMT)

To prepare the material for fecal transplantation, one fecal pellet from untreated non-MI mice was resuspended in phosphate-buffered saline (PBS) at a ratio of 35-50 mg of fecal pellet per 1 mL of PBS. For the fecal transplantation experiments, mice underwent a one-week treatment regimen with an antibiotic cocktail consisting of 0.25 mg/ml ampicillin (Cat No. SI-A9518-25G; Sigma-Aldrich, USA), 0.25 mg/ml metronidazole (Cat No. M1547-25G; Sigma-Aldrich, USA), 0.25 mg/ml neomycin (Cat No. N1876-25G; Sigma-Aldrich, USA), and 0.125 mg/ml vancomycin (Cat No. V2002-5G; Sigma-Aldrich, USA). Subsequently, the mice were colonized with 300 μl of the microbiome transplant on three occasions, administered every other day before the surgery.

### Stool DNA extraction

DNA from frozen fecal samples was extracted using the bead-beating method. Specifically, human and mouse stool DNA was extracted using the Easy-Prep Stool Genomic DNA kit (Cat No. DPT-BC28; Biotools, Taiwan), following the manufacturer’s instructions. Following the final wash step, the DNA samples were eluted with TE buffer and stored at -80° C until further analysis.

### 16S rRNA sequencing for analysis of microbiota diversity

The V3-V4 region of the 16S rRNA gene was amplified using a specific primer set (319F: 5′-CCTACGGGNGGCWGCAG-3′, 806R: 5′-GACTACHVGGGTATCTAATCC−3′) following the 16S Metagenomic Sequencing Library Preparation procedure from Illumina (USA). A total of 12.5 ng of genomic DNA was used for the PCR reaction with KAPA HiFi HotStart ReadyMix (Cat No. KR0370—v14.22; Roche, Switzerland) under the following PCR conditions: 95° C for 3 min; 25 cycles of: 95° C for 30 s, 55° C for 30 s, 72° C for 30 s; final extension at 72° C for 5 min, followed by holding at 4° C. The PCR products were visualized on a 1.5% agarose gel, and those with a prominent main strip at 500 base pairs were purified using AMPure XP beads (Cat No. A63882; Beckman Coulter, USA). For library preparation, dual indices and Illumina sequencing adapters were added to the 16S rRNA V3-V4 PCR amplicons using the Nextera XT Index Kit (Cat No. FC-131-1096; Illumina, USA). The quality of the indexed PCR products was assessed using the Qubit 4.0 Fluorometer (Thermo Fisher Scientific, USA) and the Qsep100TM system. An equal amount of the indexed PCR products was pooled and sequenced on an Illumina MiSeq platform (Illumina, USA) to generate paired 300 base pair reads. The sequence results underwent processing using QIIME 2 (version 2020.11). Primer-trimmed sequences were clustered into amplicon sequence variants (ASVs) using the q2-dada2 plugin, and sequences with ambiguous bases and chimeras were filtered out. Phylogenetic information was obtained by classifying each representative sequence with a pre-trained Naive Bayes classifier (Silva database v.138) using the qiime-feature-classifier function. Analysis of taxonomic diversity and LEfSe (Linear Discriminant Analysis Effect Size) were performed with the Galaxy platform [38] and MicrobiomeAnalyst [39]. Samples were further selected for whole-genome shotgun sequencing based on the algorithm of Microbiomes: Picking Interesting Taxa for Analysis (microPITA) [40], following criteria that considered diversity, features, and representativeness but excluded extreme cases.

### Histology examination

Paraffin-embedded tissue sections were subjected to a series of steps including dewaxing and rehydration, followed by staining using appropriate protocols. To determine the infarct size, heart sections were stained with picrosirius red for 1 hour. The stained slides were then washed with tap water three times. After staining, the sections were dehydrated, cleared with xylene, and mounted using a resinous medium (SUB-X-MOUNTING-MEDIUM; Leica Surgipath, USA). For assessing intestinal pathophysiology, rehydrated gut sections were initially stained with mouse anti-claudin-5 (Cat No.34-1600, Thermo Fisher Scientific, USA) or rabbit anti-IL17A (Cat No. GTX133781, GeneTex, USA) antibodies. Anti-mouse IgG antibodies conjugated with Alexa 488 (Cat No. A-11004; Invitrogen, USA) were utilized to detect the signal from anti-claudin-5 antibody. Anti-rabbit IgG antibodies conjugated with horseradish peroxidase (HRP) (Cat No. SA00001-7L, Proteintech, USA) were used to detect the signal from anti-IL17A antibody. Images were captured using either the LSM700 confocal microscope (Carl Zeiss, Germany) or the Pannoramic 250 FLASH II (3DHISTECH, Hungary), and quantitative analysis was performed using ImageJ software. Adjustments in brightness and contrast were applied consistently to all immunofluorescence images in a series to enhance visual clarity.

### LC-MS untargeted metabolic profiling

Hearts were obtained from both young and aged mice. After removal of the atria and aorta, the samples were rapidly frozen in liquid nitrogen and subsequently prepared for LC-MS metabolic profiling. The entire profiling process, including sample preparation, followed a previously published procedure [41].

### ^1^H-NMR metabolite profiling

The centrifugal filters (Amicon Ultra 0.5, MWCO 3 KDa; Merck, Germany) were washed three times with deionized and distilled water at 13,800×g for 20 minutes. Subsequently, 300 μl of human plasma samples were filtered through the centrifugal filter at 13,800×g for 90 minutes. The filtrates were then mixed with 100 μl of phosphate buffer (77.4 mM NaH_2_PO_4_, Cat No. S5011-500G; 22.6 mM Na_2_HPO_4_, Cat No. 255793-10G; Sigma-Aldrich, USA) in D_2_O (Cat No. AL-151882-100G; Sigma-Aldrich, USA) containing 100 μM 3-(Trimethylsilyl) propionic-2,2,3,3-d4 acid (TSP; Cat No. 269913-1G; Sigma-Aldrich, USA). The solution was adjusted to a final volume of 600 μl with phosphate buffer in D_2_O and transferred to 5 mm NMR tubes (OPTIMA, Japan) for NMR analysis. One-dimensional ^1^H-NMR spectra with water pre-saturation were recorded for all samples at 298 K on a BRUKER AVANCE III 600 MHz spectrometer equipped with a TXI (1H/13C/15N) 5 mm CryoProbe (Bruker, USA). Each spectrum was obtained with 128 scans, using a recycle delay (d1) of 2 seconds, and was processed with Topspin 2.1 (Bruker, USA). Metabolite annotation and quantification were performed using Chenomx NMR suite 8.5 (Chenomx Inc., Canada).

### Statistical analysis

Statistical analysis and graph generation were conducted using GraphPad Prism 9 (GraphPad Software, Inc., La Jolla, CA, USA). Results are presented as mean±SEM. The Kruskal–Wallis test was used for group analysis, while a two-sided Student’s t-test was employed to analyze two independent groups. Survival rate was assessed using the Kaplan–Meier method and compared using Mantel–Cox log rank tests.

## Supplementary Material

Supplementary Figure 1
