# Supplementary material for: Young microbiome transplantation enhances recovery after myocardial infarction
Source: Aging (Albany NY). 2025 Jul 15;17(7):1852–67. doi: 10.18632/aging.206279 (PMC12339018; doi:10.18632/aging.206279)
Supplement: Supplementary Figure 1 [file aging-17-206279-s001.pdf]

SUPPLEMENTARY FIGURE

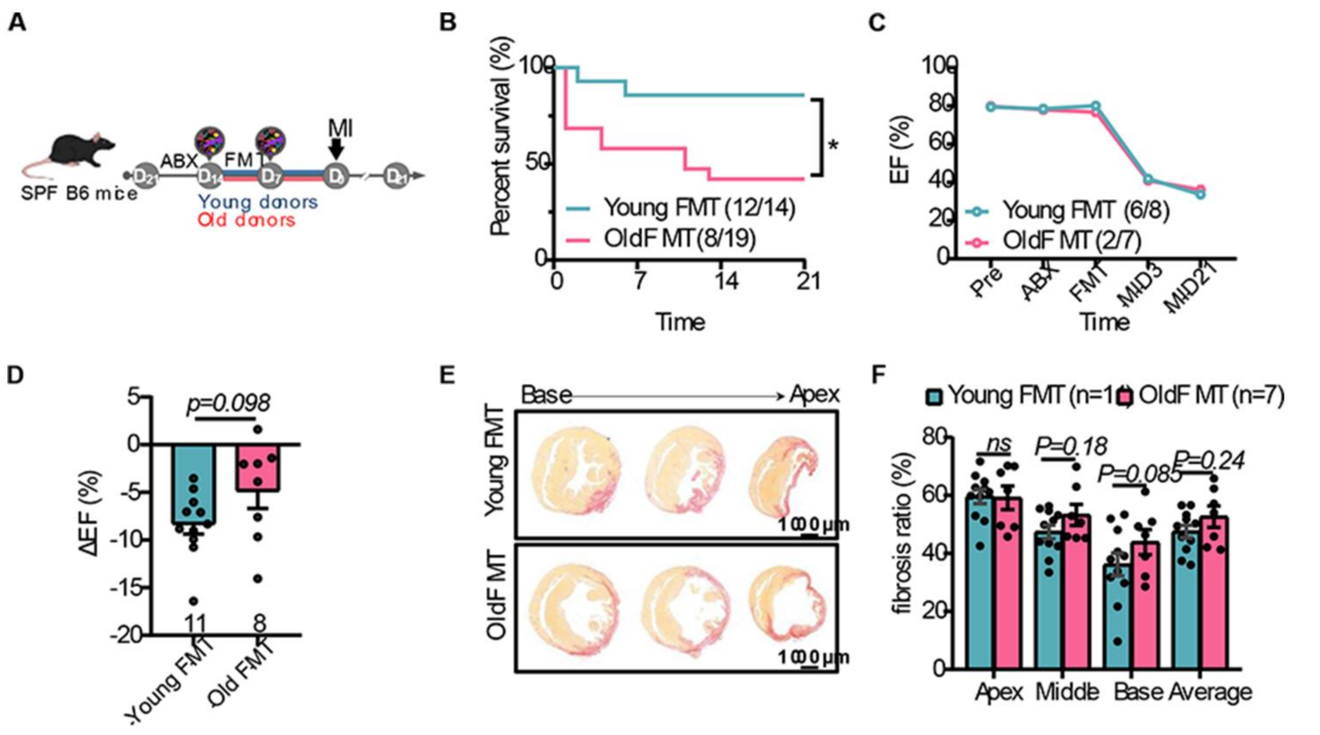

Supplementary Figure 1. Impact of aged fecal microbiome transplantation (FMT) on cardiac function in recipient male mice. (**A**) Male specific pathogen-free (SPF) mice were administrated with antibiotics (ABX) and subsequently subjected to FMT from young and aged mice prior myocardial infarction (MI). (**B**) The post-MI survival curve for male mice subjected to FMT from young and aged mice. (**C**) Cardiac ejection fraction (EF, %) of male mice before and after FMT and MI. (**D**) Quantification of cardiac ejection fraction changes (EF, %) in male mice receiving FMT from young or aged donors. (**E**) Picrosirius red staining for post-MI cardiac fibrosis of male mice receiving FMT. (**F**) Statistical analysis of post-MI cardiac fibrosis of female mice receiving FMT. Two-way ANOVA with Tukey post-hoc test was used to analyze data in (**C**, **D**, **F**) Data are represented as mean ± SEM.
